# Supplementary material for: Unraveling the Fungal Community Associated with Leaf Spot on Crataegus sp
Source: Microorganisms. 2020 Mar 24;8(3):459. doi: 10.3390/microorganisms8030459 (PMC7144009; doi:10.3390/microorganisms8030459)
Supplement: Supplementary file 1 [file microorganisms-08-00459-s001.pdf]

Supplementary:

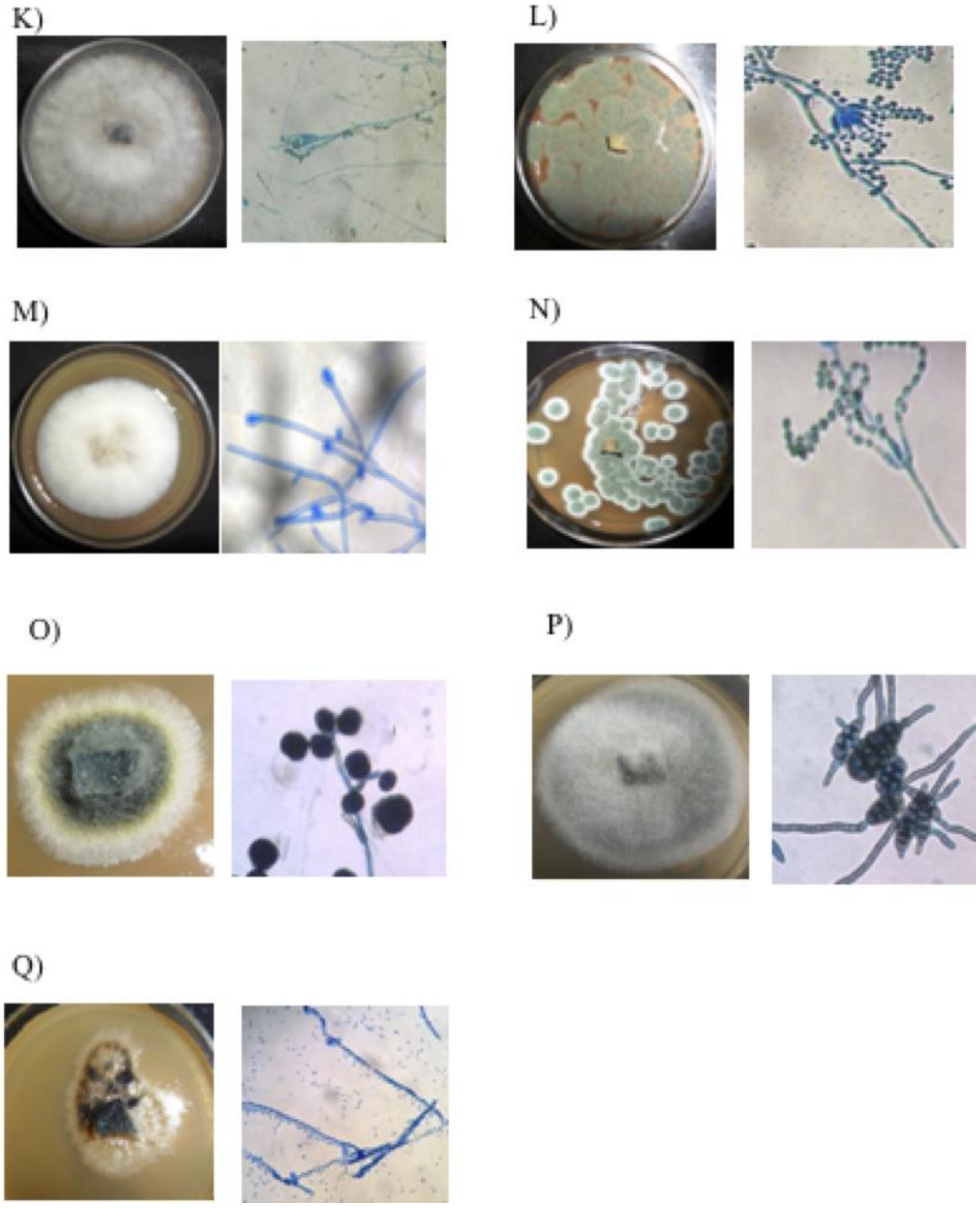

Figure S1. Phenotypical identification of the fungal isolates. In each case, the macroscopic phenotype is shown on the left and the result of the staining with cotton blue on the right, showing the microscopic features. Macroscopic and microscopic characteristics of the genera A) *Alternaria*. B) *Acremonium*, C) *Aspergillus*, D) *Aureobasidium*, E) *Cephalosporium*, F) *Drechslera*, G) *Fonsecaea*, H) *Fusarium*, I) *Geotrichum*, J) *Helmintosporium*, K) *Paecilomyces*, L) *Penicillium*, M) *Scedosporium*, N) *Scopulariopsis*, O) *Stemphylium*, P) *Ulocladium* and Q) *Wangiella* are shown.
